# Magnet-Guided Temozolomide and Ferucarbotran Loaded Nanoparticles to Enhance Therapeutic Efficacy in Glioma Model

**DOI:** 10.3390/nano14110939

**Published:** 2024-05-27

**Authors:** Reju George Thomas, Subin Kim, Thi-Anh-Thuy Tran, Young Hee Kim, Raveena Nagareddy, Tae-Young Jung, Seul Kee Kim, Yong Yeon Jeong

**Affiliations:** 1 Department of Radiology, Chonnam National University Hwasun Hospital, Hwasun 58128, Republic of Korea; regeth@gmail.com (R.G.T.);; 2 Department of Biomedical Sciences, Chonnam National University Medical School, Gwangju 501190, Republic of Korea; soooo.bean@gmail.com; 3 Biomedical Sciences Graduate Program (BMSGP), Chonnam National University, Hwasun 58128, Republic of Korea; 4 Brain Tumor Research Laboratory, Chonnam National University Hwasun Hospital, Hwasun 58128, Republic of Koreayung-ty@chonnam.ac.kr (T.-Y.J.); 5 Department of Neurosurgery, Chonnam National University Hwasun Hospital, Hwasun 58128, Republic of Korea; 6 Department of Radiology, Chonnam National University Medical School, Gwangju 61469, Republic of Korea

**Keywords:** nanoparticle, glioma, theranostics, magnetic guidance

## Abstract

**Background.** The aim of the study was to synthesize liposomal nanoparticles loaded with temozolomide and ferucarbotran (LTF) and to evaluate the theranostic effect of LTF in the glioma model. **Methods.** We synthesized an LTF that could pass through the Blood Brain Barrier (BBB) and localize in brain tumor tissue with the help of magnet guidance. We examined the chemical characteristics. Cellular uptake and cytotoxicity studies were conducted in vitro. A biodistribution and tumor inhibition study was conduted using an in vivo glioma model. **Results.** The particle size and surface charge of LTF show 108 nm and −38 mV, respectively. Additionally, the presence of ferucarbotran significantly increased the contrast agent effect of glioma compared to the control group in MR imaging. Magnet-guided LTF significantly reduced the tumor size compared to control and other groups. Furthermore, compared to the control group, our results demonstrate a significant inhibition in brain tumor size and an increase in lifespan. **Conclusions.** These findings suggest that the LTF with magnetic guidance represents a novel approach to address current obstacles, such as BBB penetration of nanoparticles and drug resistance. Magnet-guided LTF is able to enhance therapeutic efficacy in mouse brain glioma.

## 1. Introduction

Glioma is a highly aggressive primary central nervous system tumor with a low survival rate [1,2]. Surgery is the standard treatment for glioma with radiation and chemotherapy. However, the highly invasive growth pattern of glioma makes it impossible to completely remove the tumor by surgical resection without impairing the patient’s brain function, which ultimately results in tumor recurrence and death of the patient [3]. Despite conventional treatment approaches, encompassing surgery, radiation, and chemotherapy, merely 3–5% of patients exhibit favorable prognoses. Furthermore, conventional chemotherapy exhibits several limitations, including suboptimal therapeutic efficacy and considerable systemic toxicity, stemming from its inadequate selectivity for malignant cells [4].

Temozolomide (TMZ) is a first-line chemotherapeutic option in the treatment of glioblastoma and functions as a DNA-alkylating agent. By methylating guanine and adenine bases, TMZ induces DNA single-strand or double-strand breaks, cell cycle arrest, and finally, cell death [5]. The anticancer effect of TMZ is achieved by its spontaneous hydrolysis into metabolite 3-methyl-(triazen-1-yl)imidazole-4-carboximide (MTIC), which subsequently decomposes into 5-aminoimidazole-4-carboxamide (AIC) and the methyl diazonium cation, both of which then alkylates DNA. TMZ, with its low molecular weight of 194 g/mol, can penetrate the BBB and be rapidly absorbed. However, the active metabolite of TMZ, MTIC, is reportedly unable to pass through cell membranes in general and the BBB in particular [6,7]. Therefore, while TMZ can cross the BBB due to its small size, its anticancer effect in the brain relies on the generation of MTIC within the brain tissue [6,8].

The BBB, which consists of microvascular endothelial cells, tight junctions, and nerves, forms a physical barrier to protect the brain tissue and maintain the environment [9]. However, it can also shield residual tumor cells that infiltrate the surrounding brain tissue and become resistant to chemotherapeutic drugs [10,11]. Thus, overcoming the BBB is a major challenge for chemotherapy in glioma treatment, and developing effective strategies to penetrate the BBB and target the tumor site is crucial for improving outcomes. There are also some limitations for TMZ, including hematological toxicity and acute cardiomyopathy, as well as poor solubility in physiological conditions that prevent drug uptake and dosage increase [12]. Additionally, the half-life of TMZ is about 1.8 h, which results in a short duration of action. This necessitates the administration of high doses of TMZ, which can cause bone marrow suppression [8,13]. To overcome these limitations, a TMZ-loaded nanoplatform has been developed to increase solubility and improve drug uptake for glioma. 

Ferucarbotran (Resovist^®^, Bayer Healthcare, Berlin, Germany) is developed by Schering AG and is the second clinically approved superparamagnetic iron oxide (SPION) developed for contrast-enhanced MR imaging of the liver. Ferucarbotran, a type of SPION coated with carboxydextran, has been used as a T2 contrast agent [14,15]. The active particles are carboxydextrane-coated SPION, with a hydrodynamic diameter ranging between 45 and 60 nm. We have used the magnetofection method to enhance the accumulation of magnetic particle-loaded liposomes with TMZ in glioma. There are studies reported where in vivo magnetofection was performed. In this particular study, transportation of Tween-SPIONs, injected via the tail vein, through the intact blood-brain barrier in rats was subjected to an external magnetic field (EMF) [16,17]. The findings suggest that the Tween-SPIONs effectively traverse the blood-brain barrier (BBB) through an active penetration mechanism, which is facilitated by electromagnetic fields (EMF). A different strategy for the focused therapy of malignancies is magnetic hyperthermia (MH), which has been clinically introduced. In MH, an alternating magnetic field (AMF) is applied to magnetic nanoparticles (MNPs), which produce heat. Magnetofection can accumulate MNP to glioma, and MH can induce hyperthermia, causing tumor reduction. As demonstrated before, the ferucarbotran employed in our investigation likewise functions as a hyperthermia-generating nanoparticle. 

In this study, we designed and synthesized a TMZ and ferucarbotran-loaded liposome to treat GL261 brain glioma. The liposome served as a nanoparticle to deliver TMZ and ferucarbotran across the BBB. By employing TMZ in conjunction with the SPION, we hypothesized that liposomal nanoparticles loaded with TMZ and ferucarbotran (LTF) have enhanced therapeutic effects as well as visualization of glioma on MR imaging. The aim of the study was to synthesize the LTF and to evaluate the theranostic effect in the glioma model.

## 2. Materials and Methods

### 2.1. Materials

The murine glioma cell line, GL261 was used for cell culture. Normal mouse fibroblast cell line (NIH3T3) and Microglial cells (BV2) were purchased from the American Type Culture Collection (ATCC, Virginia, VA, USA). 3-(4,5-dimethylthiazol-2-yl)-5-(3-carboxymethoxyphenyl)-2-(4-sulfophenyl)-2H-tetrazolium (MTS) was purchased from Promega (Promega Corporation, Wisconsin, WI, USA). Ferucarbotran (56 mg/mL) was ordered from Meito Sangyo Ltd. (Aichi, Japan). TMZ was purchased from Sigma Aldrich (Merck, Darmstadt, Germany). All other reagents were of analytical or chromatographic grade. Antibodies for immunohistochemistry were purchased from Abcam (Cambridge, UK).

### 2.2. Synthesis of Lipo-TMZ-Ferucarbotran (LTF)

Liposome was prepared using the film hydration method. Briefly 1, 2-Distearoyl-sn-glycero-3-phosphoethanolamine-Polyethylene glycol (DSPE-PEG), Dipalmitoylphosphatidylcholine (DPPC), and cholesterol were taken in a weight ratio of 1× (300 μg: 300 μg: 200 μg), 5× (1.5 mg: 1.5 mg: 1 mg), 10× (3 mg: 3 mg: 2 mg) and 15× (4.5 mg: 4.5 mg: 3 mg) and dissolved in their respective solvents (methanol for DSPE-PEG and DPPC, and chloroform for cholesterol). The mixture was vortexed for 2 min in a glass vial, and then a thin film was formed by evaporation of the solvents in a vacuum chamber at room temperature. The resulting film was hydrated with 1 mL of distilled water and heated in a glass vial for 30 min at 60 °C to promote the formation of multivesicular liposomes, which are heterogeneous in nature. To make LTF at 5×, 10× and 15× concentrations, 2.5, 5 mg, and 7.5 mg of TMZ and 3 mg, 6 mg and 9 mg of ferucarbotran were added to 1 mL of distilled water after the film hydration process, and the synthesis method was continued as per normal liposome synthesis. Lipo-TMZ (liposomal nanoparticle loaded only with TMZ) was synthesized using the same method except for the Ferucarbotran loading step. Purification was conducted using the dialysis method with Dialysis sacks MWCO 12,000 Da (Merck, Darmstadt, Germany) for 2 h.

### 2.3. Characterization of LTF

The LTF was characterized using several techniques. The hydrodynamic size and zeta potential of LTF were measured using a dynamic light scattering (Zetasizer Nano Z instrument, Malvern, UK), while the morphology of LTF was visualized using Field Emission Transmission Electron Microscopy (FE-TEM, Hitachi S3000H, Tokyo, Japan). The drug-loading and encapsulation efficiency were evaluated by thermogravimetric analyzer (TGA N-1000, Scinco, Seoul, Republic of Korea) and ICP-MS (820 ICP-MS Varian Bruker, Billerica, MA, USA) analyses, respectively. The release study was performed using a dialysis method. To do so, 3 mg/mL of LTF was dissolved in phosphate-buffered saline (PBS) and performed by oscillating shaker at a temperature of 37 °C. At different time points (0, 0.5, 1, 2, 4, 6, 8, 24, and 48 h), 1 mL samples were obtained and replaced with fresh PBS for high-performance liquid chromatography (HPLC) analysis.

### 2.4. In Vitro Study of LTF

The cytoviability study of LTF was evaluated by MTS assay in both BV2 neuronal and NIH3T3 mouse fibroblast cells. In a 96-well plate, 1 × 10^4^ cells/well were seeded and maintained in Dulbecco’s Modified Eagle Medium (DMEM) and incubated at 5% CO_2_ and temperature of 37 °C for 24 h. After 24 h, the cell culture medium was removed, and a new medium containing TMZ, Lipo-TMZ, and LTF at a concentration range of 0 to 1000 μM [TMZ] was added to each well. Following a 24-h incubation period, 20 μL of MTS reagent was added to each well, and the plate was incubated for an additional 3 h. Finally, the absorbance was measured at 490 nm using a microplate reader. 

Prussian blue staining is used in the detection of ferric iron in glioma cells. GL261 cells were plated at a density of 10^5^ cells/well in an 8-well chamber and incubated overnight. After overnight incubation, a new medium containing LTF was treated at an equivalent Liposome concentration of 100 μg/mL for 6 h. The cells were washed with PBS three times and fixed with 4% paraformaldehyde. Then, the cells were stained with 100 μL of 10% potassium ferrocyanide and 2% hydrochloric acid at a 1:1 ratio for 20 min. After washing away the staining solution, the cells were counterstained with nuclear fast red stain for 5 min and observed under a bright field microscope.

The cytotoxicity study of TMZ and LTF was evaluated by MTS assay in GL261 cells. In a 96-well plate, 1 × 10^4^ cells/well were seeded and maintained in Dulbecco’s Modified Eagle Medium (DMEM) and incubated at 5% CO_2_ and temperature of 37 °C for 24 h. After 24 h, the cell culture medium was removed, and a new medium containing TMZ and LTF at a concentration range of 0 to 1000 μM was added to each well. Neodymium (NdFeB) Disc Magnet was placed under 96 well plate for in vitro cytotoxicity analysis in GL261 cell line for 1 h and replaced with fresh media. After incubating for 24 h, 20 μL of MTS reagent was introduced into each well, and the plate was further incubated for 3 h. Subsequently, the absorbance was assessed at 490 nm utilizing a microplate reader.

For apoptosis analysis by caspase-3 assay (Caspase 3 Colorimetric Activity Assay Kit, DEVD, Chemicon, Massachusetts, MA, USA), GL261 cell was plated in a 12-well plate at a density of 5 × 10^5^ cells/well and incubated overnight. After overnight incubation, a new medium containing free TMZ (62.5 µM) and LTF was treated at an equivalent TMZ concentration of 62.5 µM for 6 h. Next, the cells were washed with pre-cooled PBS and stained with caspase reagent for 30 min in the dark. After washing away the caspase reagent solution, the cells were observed under ZOE Fluorescent Cell Imager (Bio-Rad, California, CA, USA) bright field/Rhodamine B fluorescence filter.

Fluorescein diacetate (FDA) and propidium iodide (PI) staining was utilized to distinguish viable and dead cells in the LTF-treated GL261 cell line. This staining method detects live cells with FDA and dead cells with PI. We have incubated GL261 cells at a density of 5 × 10^5^ cells/well. After 24 h, FDA/PI was added to 0.5 mL cell solution and incubated at 37 °C for 45 min. After washing away the FDA/PI solution, the cells were observed under ZOE Fluorescent Cell Imager (Bio-Rad, CA, USA) FITC/Rhodamine filter.

Ɣ-H2AX is a marker of DNA damage and repair. It is formed when the Ser-139 residue of the H2AX histone variant is phosphorylated, indicating the presence of DNA double-strand breaks. Detecting γH2AX is a highly sensitive and specific way to monitor DNA damage and resolution. Quantifying γH2AX foci is a useful tool for evaluating the effectiveness of temozolomide in causing DNA damage. GL261 cells were cultured and seeded at 5 × 10^4^ per well in 8 well Chamber. Once the cells were attached, the cells were incubated with free TMZ 62.5 µM, and LTF was treated at an equivalent TMZ concentration of 62.5 µM for 6 h. After incubation, the cells were given a media change and stained with mouse Anti-Ɣ-H2AX overnight and Anti-mouse Flamma 594 for 1 h and fixed with Gold anti-phalloidin. Imaged in Zeiss Confocal Microscopy. Quantification was conducted using Image J Version 1.54g in different cells from the same image. 

### 2.5. Release of TMZ from LTF

To conduct the drug release study, 10 mg of LTF was dispersed in 10 mL of PBS and added into a 12 kDa membrane (SpectraPor^®^ Standard Grade RC Membrane, Fisher Scientific, Massachusetts, MA, USA). The sample was incubated at 37 °C, and 1 mL of the sample was taken in triplicates at different time points and replaced with fresh PBS. At the end of the study, the samples were dehydrated by freeze drying and dispersed in 90% methanol, then analyzed by UV-Vis. 

### 2.6. Intracranial Glioma Mouse Model

The use of animals in this study was approved by the Chonnam National University Medical School Research Institutional Animal Care and Use Committee, and all procedures were conducted in accordance with the guidelines outlined in the National Institutes of Health Guide for the Care and Use of Laboratory Animals, under the approval reference CNU IACUC-H-2019-6. Six-week-old female C57BL/6 mice weighing 20–22 g were purchased from Orient-Bio (Seongnam-si, Republic of Korea). To establish the intracranial glioma model, 1 × 10^5^ GL261 cells in 5 µL of PBS were stereotactically injected into the right striatum at a rate of 1 µL/min using a Harvard Apparatus Pump 11 Elite infusion syringe via a Hamilton syringe (Holliston, MA, USA). Injection sites were estimated by the following coordinates: 1 mm posterior, 2 mm lateral from bregma, and 4 mm deep from the cortical surface.

### 2.7. In Vivo MR Imaging and LTF Accumulation by Magnetic Guidance 

GL261 cells were implanted into 6-week-old female C57BL/6 mice, and when the size of the brain tumor reached approximately 2 mm on MR imaging, the mice were divided into five groups, each receiving TMZ, Lipo-TMZ, or LTF according to a predetermined schedule. We divided the mice into five groups of four each; Group 1: control (0 mg/kg of TMZ), Group 2: free TMZ (11.3 mg/kg of TMZ), Group 3: Lipo-TMZ (11.3 mg/kg of TMZ), Group 4: LTF (magnet off) (11.3 mg/kg of TMZ) and Group 5: LTF (magnet on) (11.3 mg/kg of TMZ). All nanoparticles were administered intravenously 200 μL dose of LTF (8 mg/mL [1.4 mg of TMZ]) to each mouse four times on days 1, 2, 5, and 6. For group 5, after the LTF was injected, the animals were anesthetized with a combination of zoletil^®^ and rompun^®^ (zoletil: rompun: PBS ratio of 1 mL:0.5 mL:8.5 mL) 100 μL intraperitoneal injection in mice, and then neodymium magnets were placed on the heads of the mouse tumor models for 1 h.

MR imaging was performed using a preclinical 1T permanent magnet scanner (M7 system, Aspect Imaging, Shoham, Israel). To evaluate the contrast enhancement and tumor reduction efficacy of LTF, T2-weighted MR imaging was acquired using fast spin echo (FSE) sequences. The scan parameters T2WI included the following: echo time (TE) of 80 ms, repetition time (TR) of 7952 ms, slice thickness of 1 mm, field of view of 25 mm, 13 slices, and a matrix of 256 × 256. To evaluate the LTF contrast effect, the signal intensity was measured in the region of interest (ROI) area of the MR image to calculate the degree of contrast enhancement. In T2-weighted MR imaging, the signal intensity decreases proportionally as the contrast enhancement is increased by the LTF-loaded ferucarbotran.

### 2.8. Biodistribution of LTF

In the study, C57BL/6 mice with intracranial glioma were administered a 200 μL dose of LTF (2 mg/mL) intravenously. Following the LTF administration, a biodistribution study was conducted on the LTF (magnet-on) group. For the magnet-on group, after the injection of LTF, the mice were anesthetized using a combination of zoletil and rompun combination with 100 μL intraperitoneal injection in mice. The mice’s heads were then positioned near a neodymium magnet for a duration of 1 h. This step aimed to facilitate the localization of LTF in the desired area, specifically the tumor region. When the tumor size reached 4 mm, we administered LTF. After 24 h of LTF administration, the mice were euthanized. Major organs (Heart, liver, spleen, lung, kidney, brain) and tumor were then harvested and subjected to Aqua regia (3 parts of Hydrochloric acid and 1 part of Nitric acid) treatment to break down the tissue and extract iron. The iron content of these organs was then investigated using inductively coupled plasma mass spectrometry (ICP-MS). This analysis aimed to determine the distribution and accumulation of iron, which serves as an indicator of the presence and localization of LTF in the organs and tumor tissues.

### 2.9. Glioma Reduction and Survival Study 

Follow-up MR imaging was obtained daily until 7 days after the initial injection to observe changes in tumor volume. Tumor volume assessment using MR imaging was based on ROIs. In this study, ROIs were manually drawn on all image slices covering the majority of the tumor area. All image data were transferred in DICOM format through a picture archiving and transmission system (PACS), and tumor volumes were calculated using RadiAnt DICOM viewer (Medixant, Poland) and INFINITT PACS M6 image analysis software (Infinite Healthcare, Seoul, Republic of Korea).

The survival of the mice was tracked for 31 days, and surviving mice that were asymptomatic at the end of the trial were sacrificed. After 31 days, no mice from groups PBS, TMZ, and Lipo-TMZ survived. In LTF (MagOff) 2 mice and LTF (MagOn) 1 mouse survived after 31 days. Every mouse with a tumor was closely monitored daily and given proper care in the form of feed and water. 

### 2.10. Immunohistochemistry

To observe the cellular damage due to LTFin the harvested organs, H&E imaging was performed using an optical microscope (Olympus). TUNEL assay was performed according to the manufacturer’s instruction (DeadEnd^TM^ Fluorometric TUNEL system, Promega, USA) and visualized with a microscopy (Zeiss Confocal Microscopy, Carl Zeiss, Oberkochen, Baden-Württemberg, Germany). For Ki67 immunochemistry, tumor slides were deparaffinized and stained with a primary anti-mouse rabbit-originated Ki67 antibody (1:1000 dilution), followed by a secondary Alexa Fluor 488 goat-originated anti-rabbit antibody (1:250 dilution) and imaged using confocal microscopy (Zeiss Confocal Microscopy). The fluorescence intensities were quantified using ImageJ software from four randomly selected areas of equal size.

### 2.11. Statistical Analysis

All data represented either the mean ± standard error of the mean or mean ± standard deviation. The differences between the groups were evaluated with one-way analyses of variance (ANOVA) or Kruskal-Wallis, which was followed by Mann-Whitney U test or Tukey’s multiple comparison tests or Student’s *t*-test. A two-sided probability value (*p*) less than 0.05 was considered statistically significant. * indicates *p* < 0.05, † indicates *p* < 0.01. All measurements were taken by observers who were blinded to the individual treatments.

## 3. Results

### 3.1. Synthesis and Characterization of LTF

**Figure 1:**
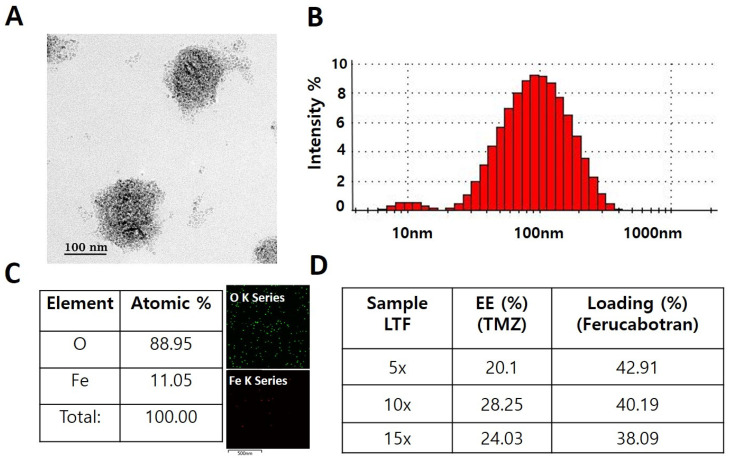
Characterization of Lipo-TMZ-Ferucarbotran. (**A**) Transmission electron microscope analysis of LTF (**B**). DLS of LTF nanoparticle. (inset figure show Size distribution from 10 nm to 1000 nm) (**C**) EDAX elemental analysis of LTF nanoparticle (**D**). Encapsulation efficiency and Thermogravimetric analysis of LTF nanoparticle.

LTF was synthesized and analyzed for size, charge, and morphological characteristics. The morphology of LTF was determined in Field emission-transmission electron microscopy (TEM) (Figure 1A). Dynamic light scattering (DLS) showed a size of 108 ± 60 nm with polydispersity index (PDI) = 0.358 and a zeta potential value of −38 mV (Figure 1B). Elemental analysis by EDAX was conducted to check the percentage of iron (Fe) and oxygen (O) in LTF.11.05% of iron and 88.95% of oxygen were detected (Figure 1C). Encapsulation efficiency (%) of TMZ was 20.1%, 28.25%, and 24.03% for 5×, 10× and 15× of LTF and thermogravimetric analysis (TGA) of LTF of 42.91%, 40.19% and 38.09% for 5×, 10× and 15× of LTF. Based on the result from EE%, we can assume that LTF 10x concentration showed the best possible encapsulation of TMZ drug at 28.25%. TGA showed 40.19% iron oxide loading of ferucarbotran in LTF 10× concentration (Figure 1D). Therefore, based on the encapsulation value of the drug and the loading amount of iron oxide, we selected LTF 10× as the candidate for in vitro and in vivo analysis.

### 3.2. In Vitro Characterization 

**Figure 2:**
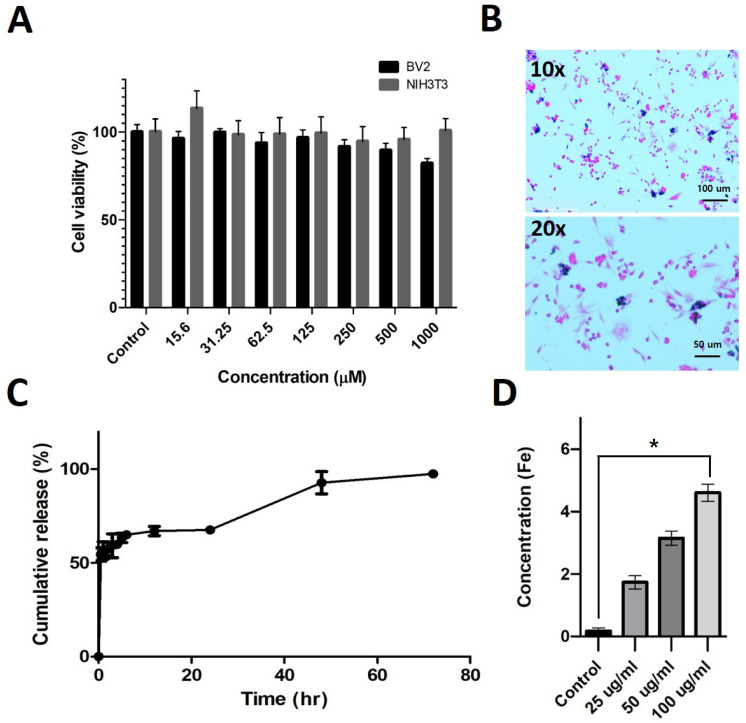
In vitro characterization of Lipo-TMZ-Ferucarbotran. (**A**) Cell viability study of Lipo-TMZ-Ferucarbotran in BV2 neuronal and NIH3T3 fibroblast cell line. (**B**) Cell uptake of Lipo-TMZ-Ferucarbotran in GL261 cell line by Prussian Blue assay. (**C**) Cumulative release profile of LTF. (**D**) ICP-AES accumulation analysis of LTF at different concentrations in GL261 cell line. The data are presented as the mean ± standard deviation control with a number of samples (* *p* < 0.05, n = 3). The Iron oxide (Fe) concentrations showed significant differences between 4 groups and in the post hoc analysis (Control vs. 25 μg/mL vs. 50 μg/mL vs. 100 μg/mL: *p* = 0.01556; Kruskal–Wallis test).

The cell viability of LTF was analyzed using the MTS assay in BV2 and NIH3T3 cell lines, with a concentration range of up to 1000 μM [TMZ] and 24-h incubation. The cell viability was maintained and exhibited minimal toxicity even at 1000 μM (Figure 2A). Cell uptake analysis was performed using Prussian blue assay (Figure 2B). Ferucarbotran, which contains iron oxide nanoparticles, reacts with acidic ferrocyanide to produce a blue color. From the cell uptake data, it is clear that LTF is taken up into the cellular compartment, providing insights into cell toxicity. 

To analyze the drug release ability of TMZ from LTF, we conducted a drug release test with UV-Vis. The data showed a burst release of 50% of TMZ within 1 h of dialysis, indicating that the liposome is susceptible to body temperature and partially breaks down to release TMZ into the bloodstream. However, 70% of the release took almost 24 h, which is longer than the brain accumulation time of LTF. The 100% release took 76 h, supporting the sustained release claim of the nanoparticle [18] (Figure 2C). A quantitative level of uptake was conducted by ICP-AES on the cell incubated with LTF. Higher concentrations of the LTF accumulated in proportion to the concentration and were statistically significantly increased at 100 ug/mL compared to the control (Figure 2D). 

**Figure 3:**
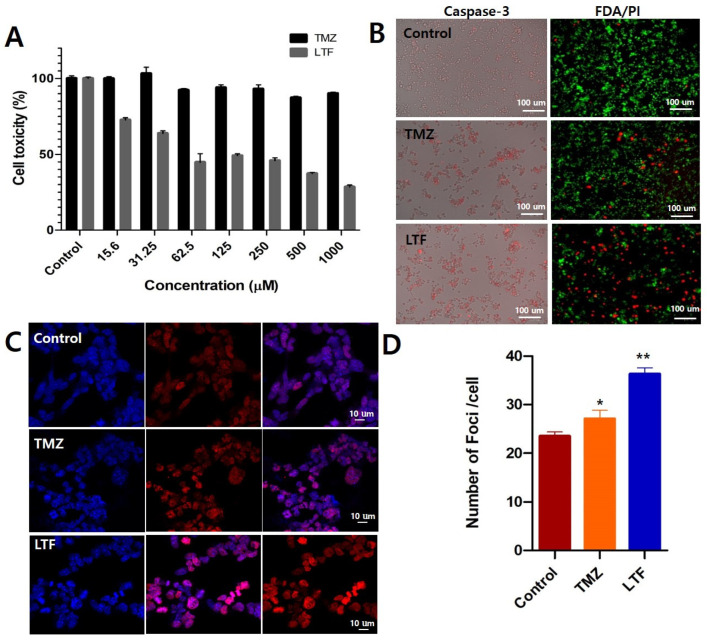
In vitro cytotoxicity of Lipo-TMZ-Ferucarbotran. (**A**) Cell toxicity study of TMZ and LTF in GL261 cell line by MTS assay with magnetofection. (**B**) Cell apoptosis study of Lipo-TMZ-Ferucarbotran in GL261 cell line by Caspase-3 assay (Red fluorescence indicates apoptotic cells). Cell death study of uptake of Lipo-TMZ-Ferucarbotran in GL261 cell line by FDA/PI staining (Green fluorescence- live cells; Red fluorescence—dead cells). (**C**) CLSM images of ɣ-H2AX (Scale bar 10 µm; Red fluorescence—double-strand break in DNA) and its (**D**) Quantification of ɣ-H2AX fluorescence. The data are presented as the mean ± standard error of the mean control (* *p* < 0.05; ** *p* < 0.01 when compared with the control group; Tukey post hoc test). The Number of Foci/Cell in the LTF group significantly increased compared with the Control or TMZ groups (Control vs. TMZ vs. LTF: *p* = 0.00001; one-way ANOVA test, TMZ vs. LTF: *p* = 0.00008; Control vs. LTF: *p* = 0.00001; Tukey post hoc test).

The cell toxicity of LTF was analyzed using the MTS assay in the GL261 cell line in comparison with free TMZ, with a concentration range of up to 1000 μM [TMZ] and Neodymium (NdFeB) Disc Magnet was placed under 96 well plate for in vitro cytotoxicity analysis in GL261 cell line for 1 h and fresh media was added. Afterward, a 24-h incubation time was given. The cell viability progressively decreased from 15.6 μM for LTF, reaching a maximum of less than 30% at a concentration of 1000 μM, whereas TMZ exhibited minimal toxicity even at 1000 μM (Figure 3A). We performed a caspase-3 assay to detect apoptosis in LTF-treated cells and found an increase in caspase-3 activity as shown by red fluorescence in the LTF group compared to cells treated with TMZ alone and the control group. Apoptosis of glioma cells is more prominent in the LTF-treated group than in the TMZ group (Figure 3B). The live/dead assay using FDA/PI showed a similar trend to the caspase 3 assay. LTF-treated cells showed more cell death, with decreased green fluorescence in FDA staining and red fluorescence in PI staining (Figure 3B). 

Double-stranded breaks (DSB) formation is an initial cellular response to radiation, and the detection of ɣ-H2AX foci serves as direct evidence to confirm the presence of DSBs. In our study, we investigated the impact of TMZ on the phosphorylation of ɣ-H2AX using CSLM imaging. As shown in (Figure 3C,D), the treated group exhibited a significant increase in the number of ɣ-H2AX foci compared to the control and TMZ group, indicating that LTF released TMZ-induced DSBs more than the TMZ group. 

### 3.3. In Vivo MR Imaging and LTF Accumulation by Magnetic Guidance 

**Figure 4:**
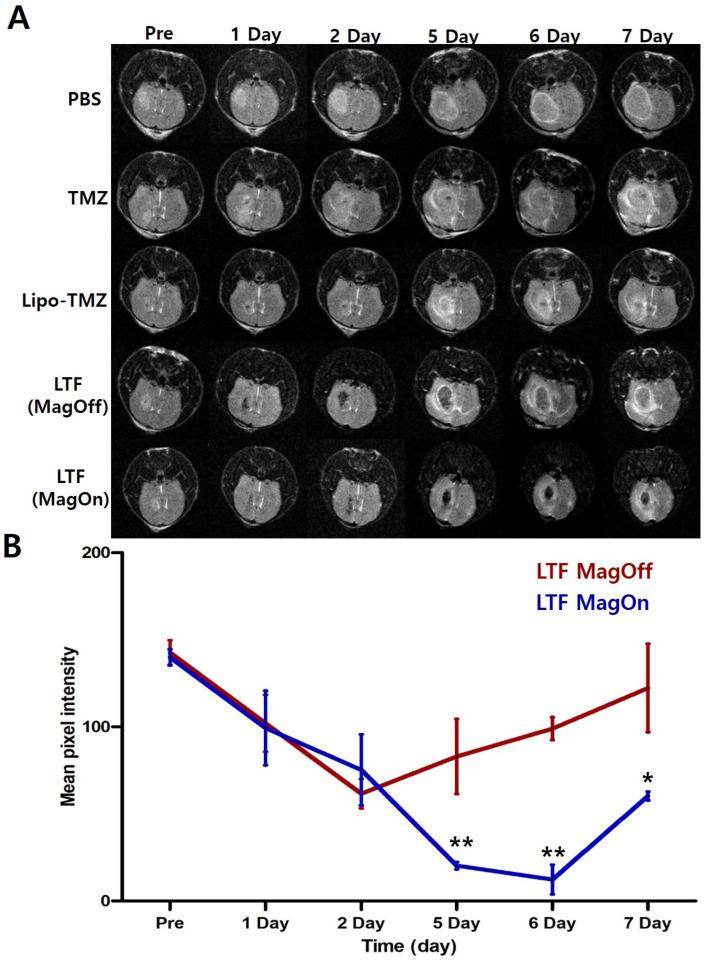
Tumor accumulation of LTF. (**A**) In vivo T2-weighted MR imaging of C57BL/6 mice injected with PBS, TMZ, Lipo-TMZ, LTF (Magnet-Off), LTF (Magnet-On) at different time points (Magnetofection was conducted for one time after injection of LTF for one hour). (**B**) A quantitative graph shows changes in MR signal intensity over time. The average pixel intensity of the MR images shows a statistically significant difference between LTF (magnet on) and LTF (magnet off) using the one-way ANOVA test. The data are presented as the mean ± standard error of the mean control (* *p* < 0.001; ** *p* < 0.01) (n = 4). The MR signal intensity showed significant differences between 2 groups at different time points (LTF (Magnet-Off) vs. LTF (Magnet-On): *p* = 0.0074 at day 5, *p* = 0.002 at day 6 and *p* = 0.0137; One-way ANOVA).

In the LTF-treated groups, the decrease in MRI signal intensity of the glioma steadily decreased over time, especially in the magnet-on group (Figure 4A). The contrast effect of LTF (magnet-on) lasted until day 6, while the contrast effect of LTF (magnet-off) decreased significantly after day 2. On days 5, 6, and 7, the LTF (magnet-on) group had a statistically significant difference in contrast effect than the magnet-off group (Figure 4B). The decrease in signal intensity indicates that more LTFs are targeted and also that more TMZ accumulates in the glioma.

### 3.4. Glioma Reduction and Survival Study 

**Figure 5:**
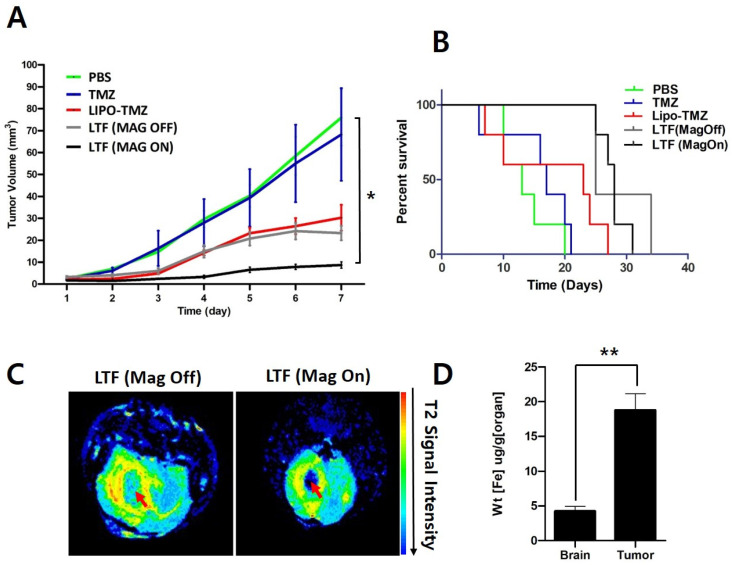
Tumor reduction study of LTF in GL261 tumor. (**A**) Tumor volume measurement after treatment of PBS, TMZ, Lipo-TMZ, LTF (mag off), and LTF (mag on). The data are presented as the mean ± standard error (* *p* < 0.5). The mean tumor size showed significant differences between the PBS, TMZ, LIPO-TMZ, LTF (MAG OFF), and LTF (MAG ON) as analyzed by the One-way ANOVA test on day 7 and Tukey post hoc test. (PBS vs. TMZ vs. LIPO-TMZ vs. LTF (MAG OFF) vs. LTF (MAG ON); One-way ANOVA: *p* = 0.002, PBS vs LTF (MAG OFF): *p* = 0.03, PBS vs. LTF (MAG ON): *p* = 0.006 and TMZ vs. LTF (MAG ON): *p* = 0.016: Tukey post hoc test). (**B**) Survival analysis of all groups after tumor reduction study was conducted using the Kaplan-Meir method. The data are presented as the mean ± standard error (* *p* < 0.5). The survival curves are significantly different between the PBS, TMZ, LIPO-TMZ, LTF (MAG OFF), and LTF (MAG ON) as analyzed by the log-rank (Mantel-Cox) test (*p* = 0.002). (**C**) MR imaging of LTF accumulation in tumor. (**D**) ICP-MS analysis of LTF accumulation in brain and tumor. The data are presented as the mean ± standard deviation with a number of samples (n) = 4. ** indicates *p* < 0.001.

Glioma growth inhibition was observed in the LTF (Magnet-On) group from day 3 to day 7, with statistical significance (* *p* < 0.01) (Figure 5A). Lipo-TMZ also demonstrated significant tumor inhibition compared to the PBS and TMZ groups, which validates the ability of liposomes to penetrate the BBB. Lipo-TMZ and LTF (magnet off) groups exhibited a similar tumor inhibition profile, as expected, since Ferucarbotran does not contribute to any chemotherapeutic effect. Survival analysis of mice showed that the median survival duration for the LTF (magnet on) group was longer than the LTF (magnet off) group, likely attributable to a decrease in tumor growth and concomitant reduction in glioma volume (Figure 5B).

In contrast, LTF (magnet on) group accumulated more than LTF (magnet off) as shown by MRI data and ICP-MS data (Figure 5C,D). 

### 3.5. Biodistribution and Immunohistochemistry

**Figure 6:**
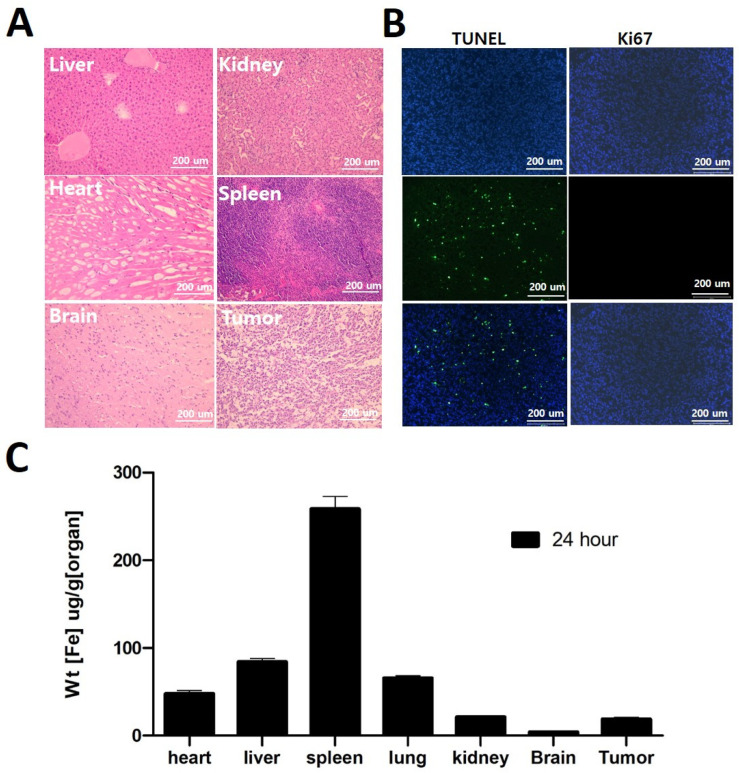
Histopathological analysis (**A**) H and E staining of tumor and organs. (**B**) TUNEL and Ki67 images of GL261 tumor extracted from brain (blue fluorescence: DAPI stain, green fluorescence: TUNEL/Ki67 stain). These findings indicate that the administration of LTF resulted in the initiation of apoptosis (cell death) in the tumor cells, accompanied by limited cell division or growth. (**C**) ICP-MS analysis of organs and tumor after 24 h of LTF administration and magnetofection.

H and E staining on the organs and tumor assessed the potential toxic effects of TMZ but found no visible toxicity, including in the brain. H and E analysis depicted in (Figure 6A) also demonstrates that, following the treatment, the regrowth of the tumor tissue occurs with a reduced density of brain tissue. This suggests that the treatment has a suppressive effect on the growth and density of the tumor, contributing to the observed survival benefits. TUNEL analysis, which detects DNA fragmentation associated with apoptosis, investigates the presence of apoptotic cells in the tumor region of the brain. The TUNEL analysis showed green fluorescence, indicating the occurrence of apoptosis in that specific area (Figure 6B).

Additionally, we employed Ki67 staining to identify proliferating cells, which are indicative of active cell division. Interestingly, the Ki67 staining did not show any proliferating cells in the tumor region, suggesting that the tumor cells had a very low proliferation index. These findings suggest that LTF treatment led to the induction of apoptosis in the tumor cells with minimal cell division or growth (Figure 6B). We can correlate H and E analysis of tumor tissue with reduced growth and density of the tumor to Ki67 staining data with a very low proliferation index and conclude that tumor growth is significantly affected by TMZ from LTF nanoparticles.

ICP-MS analysis in the LTF (magnet on) group revealed a significant accumulation of SPION in the tumor compared to the brain (Figure 6C). This suggests that the iron oxide specifically accumulated in the tumor region. Additionally, the study examined the distribution of LTF in various organs, and the results showed the highest accumulation of LTF in the spleen, which is known to serve as a storage organ for iron in the body [19]. 

Moreover, the study compared the accumulation of LTF in glioma tissue with that in normal brain tissue. The findings indicated that glioma tissue exhibited a higher accumulation of LTF than normal brain tissue, implying a potential targeting effect of LTF on the tumor. We can relate the effect of a higher accumulation of LTF nanoparticles in glioma tissue with the immunohistochemistry data, which shows that more TMZ accumulation can cause reduced tumor cell proliferation and thus inhibit glioma progression [20].

## 4. Discussion

To the best of our knowledge, this is the first report of targeted treatment of glioma under magnetic guidance by loading TMZ and SPION together in liposome nanoparticles. In our study, we successfully synthesized LTF nanoparticles loaded with TMZ and ferucarbotran and confirmed that the therapeutic effect of TMZ on glioma was increased by targeted delivery of TMZ to tumor cells. We also confirmed the contrast enhancement effect of glioma in MR imaging. 

Treatment of glioma remains a challenge, largely due to the fast degradation of TMZ, the inability to deliver an effective dose of TMZ to tumors, and the lack of target specificity, which may cause systemic toxicity. Various attempts are being made to overcome this, especially targeted therapies using various nanoparticles. Similar to our study, previous studies reported that TMZ in liposomal formulation showed enhanced uptake by glioma cells compared to free TMZ, which relies only on passive diffusion for cellular uptake. The use of liposomal nanoparticles to enhance the delivery of TMZ across the BBB and improve the therapeutic efficacy of the drug [9,21]. 

Gliomas are characterized by endothelial proliferation by angiogenesis and the formation of tortuous, disorganized, and highly permeable vessels [22]. These excessive neovessels disrupt the BBB and cause contrast enhancement of the glioma on MR imaging [23]. Contrast agents such as gadolinium and spions do not cross the healthy BBB, but in tumors such as glioma, the BBB is physically destroyed by angiogenesis, allowing the contrast agent to leak into the tumor tissue, making the tumor visible on T1-weighted MR images [24]. So, in theory, intravenously administered nanoparticles could take advantage of this phenomenon in glioma for imaging and treatment. Optimum nanoparticle formulations were chosen according to particle size, TEM imaging, and higher encapsulation efficiency. The ideal nanoparticle formulation for localized brain delivery should be in a range of 10–200 nm and is preferred to be spherical [25]. In our study, the nanoparticles were 108 nm in size and passed through the BBB, allowing us to observe changes in tumor size in MR imaging. Based on the result from encapsulation efficiency, LTF 10x concentration showed the best possible loading of ferucarbotran and TMZ in liposomes used for in vitro and in vivo studies. Cumulative release data also show sustained release of TMZ over a period of 0 to 50 h, which allows LTF to get implanted in the tumor and release TMZ.

Cytotoxicity study revealed that the cell toxicity of LTF was significantly higher than that of free TMZ. This finding implies that the LTF system could offer enhanced therapeutic efficacy compared to conventional treatment methods. Previous studies have suggested that liposomal formulations of TMZ may have better uptake by glioma cells compared to TMZ taken up by cancer cells through passive diffusion alone [26,27]. In vitro uptake quantification by ICP-OES showed a concentration-mediated uptake profile of LTF in the GL261 cell line with the highest accumulation at 100 ug/mL concentration. Qualitative analysis by Prussian blue staining also shows significant uptake of LTF in GL261 cells. 

Our study employed the LTF system, which demonstrated substantial nanoparticle accumulation in the magnetic guidance group in comparison to the non-magnetic guidance group, as evidenced by T2-weighed MR imaging. Even though BBB penetration is favored by positively charged nanoparticles, LTF exhibits negative zeta potential, which helps it to escape reticuloendothelial system (RES) phagocytosis. This result was attributed to the magnetic effect of ferucarbotran, a SPION agent. SPIONs are extensively investigated as standalone theranostic particles as well. The magnetic iron core can be visualized via MR imaging, allowing tracking of the particles and simultaneously presenting a way of directing the particles toward their goal via magnetic fields [28]. In addition, the attractive force of the external magnetic field can be utilized to guide the ferucarbotran toward the tumor site, thereby increasing the concentration of ferucarbotran in the tumor compared to the surrounding healthy tissue [29,30]. 

Our study demonstrated a significant inhibition of brain tumor size and an increase in lifespan compared to the control group. The targeted delivery of LTF to glioma is further facilitated by the application of an external magnetic field, and the therapeutic effect is further improved. These findings suggest that the LTF with magnetic guidance represents a promising approach to address current obstacles, such as BBB penetration of nanoparticles and drug resistance. The increased accumulation facilitated a significant reduction in tumor size, primarily due to the enhanced delivery of TMZ to the tumor site. 

The ability to accurately visualize and quantify nanoparticle accumulation in tumor areas can greatly improve our understanding of biodistribution. ICP-MS analysis in the LTF (magnet on) group revealed a significant accumulation of SPION in the tumor compared to the normal brain. This finding suggests that magnetic guidance plays a crucial role in enhancing LTF accumulation within the tumor, resulting in improved therapeutic effect of the chemotherapeutic agent. Future studies should focus on optimizing the LTF system and magnetic guidance for enhanced drug delivery, as well as assessing the long-term safety and efficacy of this approach in preclinical and clinical settings. 

In summary, our study showed to synthesize LTF is a promising drug delivery system for targeted glioma therapy. LTFs were loaded with TMZ and ferucarbotran so that more TMZ accumulated in glioma cells due to the magnetic effect of ferucarbotran. In vitro cytotoxicity studies showed that LTFs had a toxic effect on glioma cells. In an in vivo tumor reduction study, LTF reduced tumor size more effectively in the magnetic guidance group than TMZ or LTF without magnetic guidance.

In conclusion, this study provides a comprehensive characterization and evaluation of LTF as a targeted drug delivery system for glioma treatment. The combination of liposomal encapsulation and magnetic guidance has demonstrated improved therapeutic outcomes in a glioma mouse model. LTF may be a promising platform for further development and eventual clinical application.

## Data Availability

Data are contained within the article and Supplementary Materials.

## Supplementary Materials

The following supporting information can be downloaded at: https://www.mdpi.com/article/10.3390/nano14110939/s1, Figure S1: (A) TEM image of LTF at higher magnification (B) Prussian blue staining of LTF at higher magnification (C) DLS size distribution of Ferucarbotran. Figure S2: Size of LTF at Day 1, Day 7 and Day 14. Figure S3: Cell viability study of Lipo-Ferucarbotran in NIH3T3 fibroblast cell line. Figure S4: Positioning of Neodymium (NdFeB) Disc Magnet (10 mm diameter and 5 mm thick) for in vitro and in vivo studies (A) Neodymium (NdFeB) Disc Magnet was placed under 96 well plate for in vitro cytotoxicity analysis in GL261 cell line (B) Neodymium (NdFeB) Disc Magnet was placed at axial plane relative to mice brain. Table S1: Size and Zeta potential values of LTF at 5×, 10× and 15× concentration.

## References

[1] Lee S.Y. (2016). Temozolomide resistance in glioblastoma multiforme. Genes Dis..

[2] Stupp R., Mason W.P., van den Bent M.J., Weller M., Fisher B., Taphoorn M.J., Belanger K. (2005). Radiotherapy plus concomitant and adjuvant temozolomide for glioblastoma. N. Engl. J. Med..

[3] Giese A., Bjerkvig R., Berens M.E., Westphal M. (2003). Cost of migration: Invasion of malignant gliomas and implications for treatment. J. Clin. Oncol. Off. J. Am. Soc. Clin. Oncol..

[4] Hoda M. (2021). Potential Alternatives to Conventional Cancer Therapeutic Approaches: The Way Forward. Curr. Pharm. Biotechnol..

[5] Strobel H., Baisch T., Fitzel R., Schilberg K., Siegelin M.D., Karpel-Massler G., Debatin K.-M., Westhoff M.-A. (2019). Temozolomide and Other Alkylating Agents in Glioblastoma Therapy. Biomedicines.

[6] Newlands E.S., Blackledge G.R., Slack J.A., Rustin G.J., Smith D.B., Stuart N.S., Quarterman C.P., Hoffman R., Stevens M.F.G., Brampton M.H. (1992). Phase I trial of temozolomide (CCRG 81045: M&B 39831: NSC 362856). Br. J. Cancer.

[7] Baker S.D., Wirth M., Statkevich P., Reidenberg P., Alton K., Sartorius S.E., Dugan M., Cutler D., Batra V., Grochow L.B. (1999). Absorption, metabolism, and excretion of 14C-temozolomide following oral administration to patients with advanced cancer. Clin. Cancer Res. Off. J. Am. Assoc. Cancer Res..

[8] Lam F.C., Morton S.W., Wyckoff J., Vu Han T.L., Hwang M.K., Maffa A., Balkanska-Sinclair E., Yaffe M.B., Floyd S.R., Hammond P.T. (2018). Enhanced efficacy of combined temozolomide and bromodomain inhibitor therapy for gliomas using targeted nanoparticles. Nat. Commun..

[9] Zhao M., van Straten D., Broekman M.L.D., Préat V., Schiffelers R.M. (2020). Nanocarrier-based drug combination therapy for glioblastoma. Theranostics.

[10] Gregory J.V., Kadiyala P., Doherty R., Cadena M., Habeel S., Ruoslahti E., Lowenstein P.R., Castro M.G., Lahhan J. (2020). Systemic brain tumor delivery of synthetic protein nanoparticles for glioblastoma therapy. Nat. Commun..

[11] Fang C., Wang K., Stephen Z.R., Mu Q., Kievit F.M., Chiu D.T., Press O.W., Zhang M. (2015). Temozolomide nanoparticles for targeted glioblastoma therapy. ACS Appl. Mater. Interfaces.

[12] Nordling-David M.M., Yaffe R., Guez D., Meirow H., Last D., Grad E., Salomon S., Sharabi S., Levi-Kalisman Y., Golomb G. (2017). Liposomal temozolomide drug delivery using convection enhanced delivery. J. Control. Release Off. J. Control. Release Soc..

[13] Jiang G., Li R., Tang J., Ma Y., Hou X., Yang C., Guo W., Xin Y., Liu Y. (2017). Formulation of temozolomide-loaded nanoparticles and their targeting potential to melanoma cells. Oncol. Rep..

[14] Chung T.-H., Hsiao J.-K., Yao M., Hsu S.-C., Liu H.-M., Huang D.-M. (2015). Ferucarbotran, a carboxydextran-coated superparamagnetic iron oxide nanoparticle, induces endosomal recycling, contributing to cellular and exosomal EGFR overexpression for cancer therapy. RSC Adv..

[15] Hamm B., Staks T., Taupitz M., Maibauer R., Speidel A., Huppertz A., Frenzel T., Lawaczeck R., Wolf K.J., Lange L. (1994). Contrast-enhanced MR imaging of liver and spleen: First experience in humans with a new superparamagnetic iron oxide. J. Magn. Reson. Imaging.

[16] Huang Y., Zhang B., Xie S., Yang B., Xu Q., Tan J. (2016). Superparamagnetic Iron Oxide Nanoparticles Modified with Tween 80 Pass through the Intact Blood–Brain Barrier in Rats under Magnetic Field. ACS Appl. Mater. Interfaces.

[17] Thomsen L.B., Thomsen M.S., Moos T. (2015). Targeted drug delivery to the brain using magnetic nanoparticles. Therapeutic Delivery.

[18] Yoo J., Won Y.-Y. (2020). Phenomenology of the Initial Burst Release of Drugs from PLGA Microparticles. ACS Biomater. Sci. Eng..

[19] Kolnagou A., Michaelides Y., Kontoghiorghe C.N., Kontoghiorghes G.J. (2013). The importance of spleen, spleen iron, and splenectomy for determining total body iron load, ferrikinetics, and iron toxicity in thalassemia major patients. Toxicol. Mech. Methods.

[20] Erthal L.C.S., Shi Y., Sweeney K.J., Gobbo O.L., Ruiz-Hernandez E. (2023). Nanocomposite formulation for a sustained release of free drug and drug-loaded responsive nanoparticles: An approach for a local therapy of glioblastoma multiforme. Sci. Rep..

[21] Amarandi R.M., Ibanescu A., Carasevici E., Marin L., Dragoi B. (2022). Liposomal-Based Formulations: A Path from Basic Research to Temozolomide Delivery Inside Glioblastoma Tissue. Pharmaceutics.

[22] Dubois L.G., Campanati L., Righy C., D’Andrea-Meira I., Leite de Sampaio e Spohr T.C., Porto-Carreiro I., Pereira C.M., Balca-Silva J., Kahn S.A., DosSantos M.F. (2014). Gliomas and the vascular fragility of the blood brain barrier. Front. Cell. Neurosci..

[23] Holodny A.I., Nusbaum A.O., Festa S., Pronin I.N., Lee H.J., Kalnin A.J. (1999). Correlation between the degree of contrast enhancement and the volume of peritumoral edema in meningiomas and malignant gliomas. Neuroradiology.

[24] Heye A.K., Culling R.D., del C. Valdés Hernández M., Thrippleton M.J., Wardlaw J.M. (2014). Assessment of blood-brain barrier disruption using dynamic contrast-enhanced MRI. A systematic review. NeuroImage Clin..

[25] Brown T.D., Habibi N., Wu D., Lahann J., Mitragotri S. (2020). Effect of Nanoparticle Composition, Size, Shape, and Stiffness on Penetration Across the Blood–Brain Barrier. ACS Biomater. Sci. Eng..

[26] Zou Y., Wang Y., Xu S., Liu Y., Yin J., Lovejoy D.B., Zheng M., Liang X.-J., Park J.B., Efremov Y.M. (2022). Brain Co-Delivery of Temozolomide and Cisplatin for Combinatorial Glioblastoma Chemotherapy. Adv. Mater..

[27] Barenholz Y. (2001). Liposome application: Problems and prospects. Curr. Opin. Colloid Interface Sci..

[28] Gobbo O.L., Sjaastad K., Radomski M.W., Volkov Y., Prina-Mello A. (2015). Magnetic Nanoparticles in Cancer Theranostics. Theranostics.

[29] Kritika, Roy I. (2022). Therapeutic applications of magnetic nanoparticles: Recent advances. Mater. Adv..

[30] Ravikanth R. (2017). Advanced Magnetic Resonance Imaging of Glioblastoma Multiforme. J. Neurosci. Rural Pract..

