# Supplementary material for: Magnet-Guided Temozolomide and Ferucarbotran Loaded Nanoparticles to Enhance Therapeutic Efficacy in Glioma Model
Source: Nanomaterials (Basel). 2024 May 27;14(11):939. doi: 10.3390/nano14110939 (PMC11173836; doi:10.3390/nano14110939)
Supplement: Supplementary file 1 [file nanomaterials-14-00939-s001.zip › nanomaterials-3007323-supplementary.pdf]

Supplementary Information

Magnet-Guided Temozolomide and Ferucarbotran Loaded Nanoparticles to Enhance Therapeutic Efficacy in Glioma Model

Reju George Thomas [1,†], Subin Kim [2,†], Thi-Anh-Thuy Tran [3,4], Young Hee Kim [4], Raveena Nagareddy [1], Tae-Young Jung [4,5], Seul Kee Kim [1,6,*] and Yong Yeon Jeong [1,6,*]

[1] Department of Radiology, Chonnam National University Hwasun Hospital, Hwasun 58128, Republic of Korea; regeth@gmail.com (R.G.T.)
[2] Department of Biomedical Sciences, Chonnam National University Medical School, Gwangju 501190, Republic of Korea; soooo.bean@gmail.com
[3] Biomedical Sciences Graduate Program (BMSGP), Chonnam National University, Hwasun 58128, Republic of Korea
[4] Brain Tumor Research Laboratory, Chonnam National University Hwasun Hospital, Hwasun 58128, Republic of Korea; yung-ty@chonnam.ac.kr (T.-Y.J.)
[5] Department of Neurosurgery, Chonnam National University Hwasun Hospital, Hwasun 58128, Republic of Korea
[6] Department of Radiology, Chonnam National University Medical School, Gwangju 61469, Republic of Korea
* Correspondence: kimsk.rad@gmail.com (S.K.K.); yjeong@jnu.ac.kr (Y.Y.J.)
† These authors contributed equally to this work.

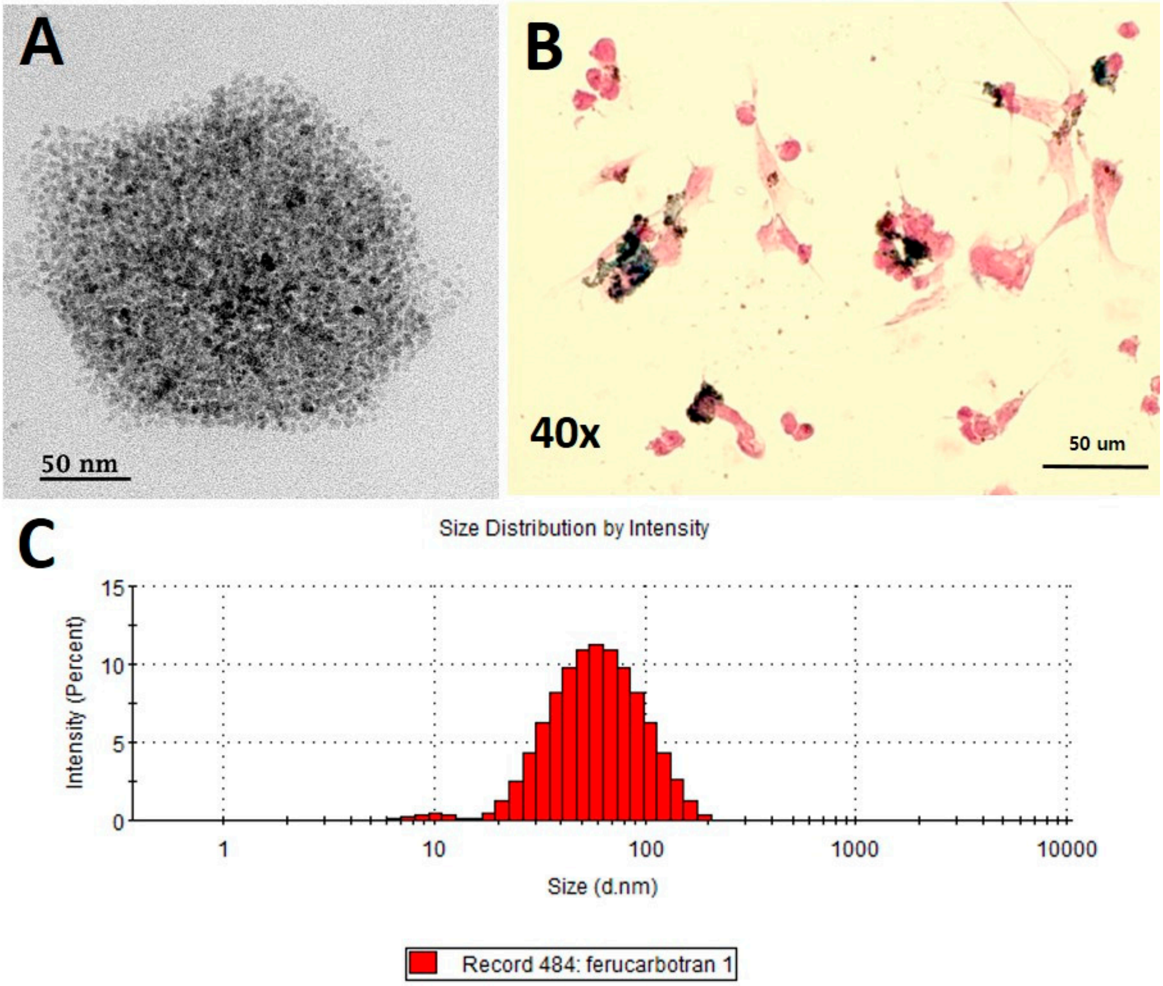

Figure S1: (A) TEM image of LTF at higher magnification (B) Prussian blue staining of LTF at higher magnification (C) DLS size distribution of Ferucarbotran.

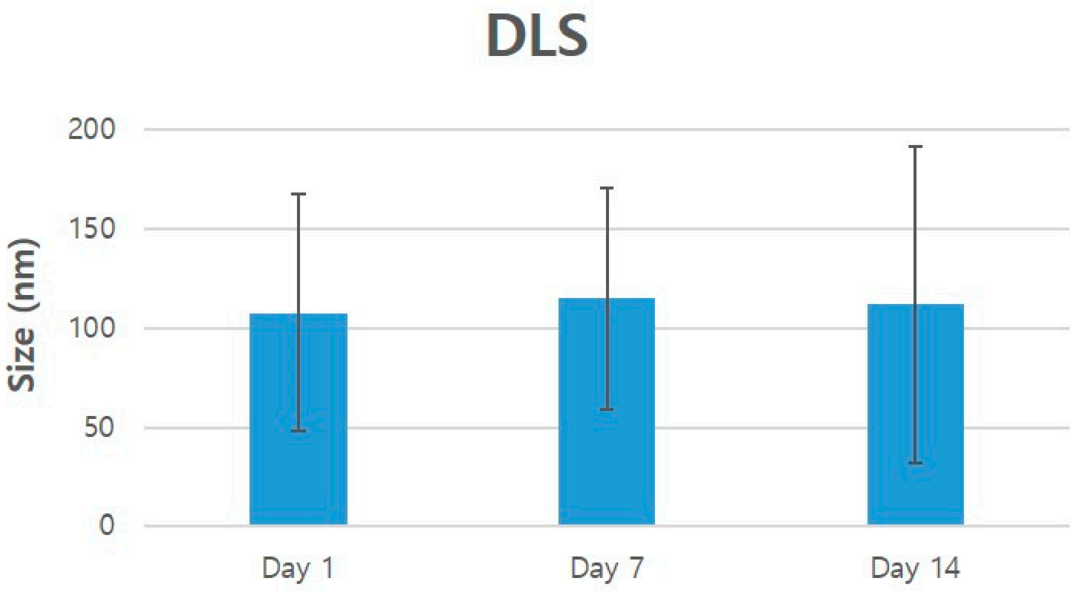

Figure S2: Size of LTF at Day 1, Day 7 and Day 14.

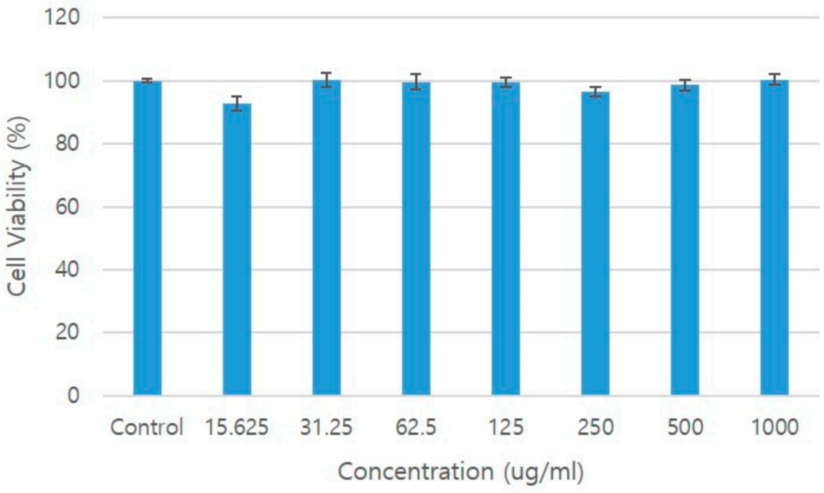

Figure S3: Cell viability study of Lipo-Ferucarbotran in NIH3T3 fibroblast cell line.

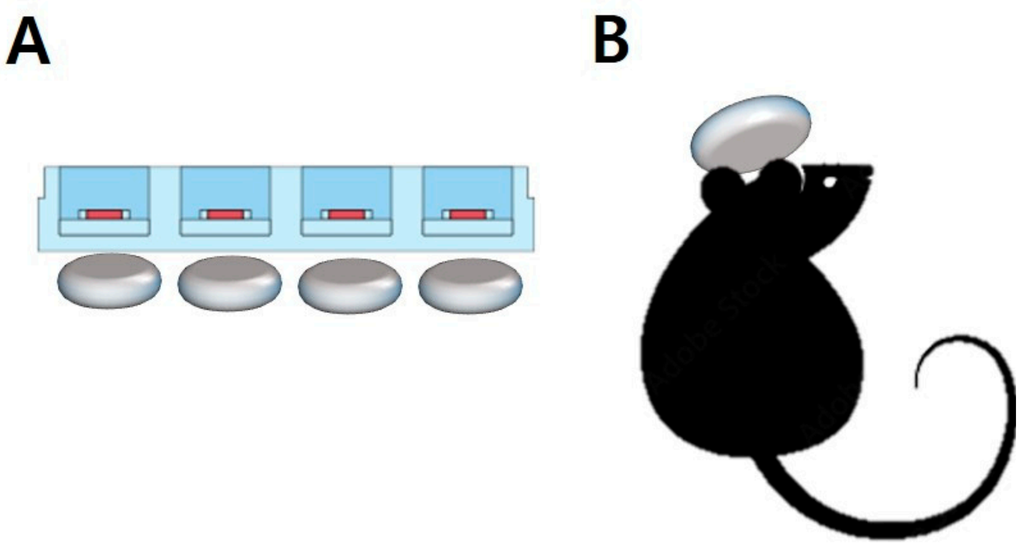

Figure S4: Positioning of Neodymium (NdFeB) Disc Magnet (10 mm diameter and 5 mm thick) for in vitro and in vivo studies (A) Neodymium (NdFeB) Disc Magnet was placed under 96 well plate for in vitro cytotoxicity analysis in GL261 cell line (B) Neodymium (NdFeB) Disc Magnet was placed at axial plane relative to mice brain.

Concentration of LTF	DLS (nm)	Zeta (charge)
5x	106±54	-50±5
10x	108±60	-36±7
15x	126±71	-40±7

Table S1: Size and Zeta potential values of LTF at 5x, 10x and 15x concentration.
